# Homology-based identification of candidate genes for male sterility editing in upland cotton (*Gossypium hirsutum* L.)

**DOI:** 10.3389/fpls.2022.1006264

**Published:** 2022-12-14

**Authors:** Karina Y. Morales, Aya H. Bridgeland, Kater D. Hake, Joshua A. Udall, Michael J. Thomson, John Z. Yu

**Affiliations:** ^1^ USDA-ARS, Southern Plains Agricultural Research Center, College Station, TX, United States; ^2^ Department of Soil and Crop Sciences, Texas A&M University, College Station, TX, United States; ^3^ Cotton Incorporated, Agricultural and Environment Research, Cary, NC, United States

**Keywords:** upland cotton, candidate genes, heterosis, homology, genetic male sterility, genome editing

## Abstract

Upland cotton (*Gossypium hirsutum* L.) accounts for more than 90% of the world’s cotton production, providing natural material for the textile and oilseed industries worldwide. One strategy for improving upland cotton yields is through increased adoption of hybrids; however, emasculation of cotton flowers is incredibly time-consuming and genetic sources of cotton male sterility are limited. Here we review the known biochemical modes of plant nuclear male sterility (NMS), often known as plant genetic male sterility (GMS), and characterized them into four groups: transcriptional regulation, splicing, fatty acid transport and processing, and sugar transport and processing. We have explored protein sequence homology from 30 GMS genes of three monocots (maize, rice, and wheat) and three dicots (Arabidopsis, soybean, and tomato). We have analyzed evolutionary relationships between monocot and dicot GMS genes to describe the relative similarity and relatedness of these genes identified. Five were lowly conserved to their source species, four unique to monocots, five unique to dicots, 14 highly conserved among all species, and two in the other category. Using this source, we have identified 23 potential candidate genes within the upland cotton genome for the development of new male sterile germplasm to be used in hybrid cotton breeding. Combining homology-based studies with genome editing may allow for the discovery and validation of GMS genes that previously had no diversity observed in cotton and may allow for development of a desirable male sterile mutant to be used in hybrid cotton production.

## Introduction

Heterosis, or hybrid vigor, is defined as the superior performance of a hybrid offspring over its inbred parents. Many plant species exhibit heterosis, including upland cotton (*Gossypium hirsutum* L.), the leading crop for the production of natural fibers used in the textile industry (Voora et al., 2020) and an important oilseed crop for human food and animal feed (Kohel and Yu, 2007). To produce hybrid seeds, an inbred male plant is crossed with an inbred female plant that has been prevented from self-pollinating, thus producing only hybrid progeny. In most plants, pollen control in the female parent can be accomplished through hand emasculation, but this is a time and labor-consuming task that cannot be scaled to the demands of commercial agriculture. As an alternative to emasculation, genetic male sterility can be used for hybrid seed production, but sources of genetic male sterility in upland cotton are limited. At this time, only a small number of genes associated with male reproductive function have been identified in cotton (summarized in **Supplementary Table 1**), despite studies in other species that have established that large numbers of genes are involved in the process of anther formation and microsporogenesis (Richmond and Kohel, 1961; Allison and Fisher, 1964; Ferrario et al., 2004; Ma, 2005; Wang and Li, 2009; Chang et al., 2011; Li et al., 2013; Wei et al., 2013; Wei et al., 2013; Wu et al., 2015; Wang et al., 2019; Li et al., 2020; Ma et al., 2021, Zhang et al., 2021a). Of these previously identified cotton genes potentially for male reproductivity and/or sterility, many are not mapped or have no known protein sequence available, thus making it difficult to manipulate them *via* methods such as genetic engineering or genome editing. For example, between the 1960s to 1990s, over 10 genes associated with genetic male sterility were identified in upland cotton (**Supplementary Table 1**), but only recently have three of these genes, *ms15, ms5*, and *ms6*, been mapped (Justus and Leinwebr, 1960; Richmond and Kohel, 1961; Justus et al., 1963; Allison and Fisher, 1964; Weaver, 1968; Weaver and Ashley, 1971; Bowman and Weaver, 1979; Huang and Shi, 1988; Zhang et al., 1992; Chen et al., 2009). These factors hinder the development and utilization of hybrid cotton in mechanized agriculture.

Male sterility arises from the abortion or malformation of male gametes, but usually does not affect female gametes, effectively creating a female parent that lacks the ability to self-pollinate (Kun et al., 2013). Classically, pollen has been described as containing four microspores, or pollen sacs, with each of these containing cells which will develop into male gametophytes, surrounded by a tapetal layer, layer(s) of parietal tissue, an epidermal layer, and a connective tissue which will hold these four pollen sacs together as a grouping within the anther (Horner and Palmer, 1995). Formation of the tapetum is a critical part of anther development, as the tapetum will collect resources from vascular tissue to redistribute to developing microspores (Pacini, 2010). Premature cell death of tapetal cells prevents the viability of pollen cells and is controlled by a variety of genes including *Baymax1*, *Carbon Starved Anther*, *Aborted Microspores*, and *AtMs1* (Sorensen et al., 2003; Zhang et al., 2010; Zhang et al., 2013; Li et al., 2016; Zhang et al., 2018; An et al., 2020; Xiang et al., 2021; Li et al., 2022). Traditionally, anther development is divided into 10 stages with meiosis occurring in stage 1 and 2, followed by mitotic divisions to form tetrads in stages 3-4. Microspore development then occurs in stages 5-7 with pollen development completing in stages 8-10 (Horner and Palmer, 1995). Abnormal development at any of these stages may lead to male sterility with mutations earlier in the developmental stages often having a larger impact.

The genetic control of male sterility can occur *via* cytoplasmic male sterility (CMS) or nuclear male sterility (NMS), also known as genetic male sterility (GMS), systems. CMS is controlled by the interaction of gene products between mitochondrial and nuclear genomes. In hybrid breeding, a three-line system is used that involves the use of a CMS line, a male-fertile restorer line, and a fertile sterility-maintainer line that is isogenic to the CMS line. The three-line system includes two crosses. To produce fertile F1 hybrid planting seed, the restorer line (used as the male parent) is crossed with the CMS line (female parent). To continue to produce CMS seed, the isogenic maintainer line (male parent) is crossed with the CMS line (female parent), which ensures that the seeds from the cross are CMS while preserving agronomically beneficial traits. CMS is stable across environments, leading to widespread historical use in some species such as rice and maize, but some types of cytoplasm are known to spontaneously revert and become fertile to varying degrees under specific environmental conditions, and fertility restoration is not always reliable (Havey, 2004; Weider et al., 2009; Chen and Liu, 2014; Abbas et al., 2021; Zhang et al., 2022). As of a 2014 review by Chen and Liu, 28 CMS genes have been identified across 13 crops (Chen and Liu, 2014). Within upland cotton, some CMS markers have been developed for breeding purposes, while transcriptomic and metabolomic studies have identified some interesting differentially expressed genes for further study of this trait (**Supplementary Table 1**) (Jiang et al., 2007; Wang et al., 2010; Wu et al., 2011; Chen and Liu, 2014; Yang et al., 2014; Kong et al., 2017; Li et al., 2018; Nie et al., 2018; Li et al., 2019; Li et al., 2021; You et al., 2022). At this time, identifying the specific mechanisms behind CMS in cotton is still an area that is under development; most recently, Zhang, et al. identified and mapped *orf610a*, a mitochondrial chimeric gene, as one causal gene of CMS in cotton (**Supplementary Table 1**) (Zhang et al., 2022).

Environment-sensitive genetic male sterility (EGMS) contrasts with CMS as its fertility is controlled by known environmental conditions such as temperature (TGMS), photoperiod (PGMS), humidity (HGMS), or a combination of temperature and photoperiod (TPGMS) (Min et al., 2013; Chen and Liu, 2014; Chang et al., 2016; Ding et al., 2017; Zhang et al., 2020; Ma et al., 2021, Zhang et al., 2021b, Li et al., 2022). Under EGMS, a male sterile line can revert to fully fertile depending on the environment. For example, the same line can be male sterile at high temperature while having full fertility at low temperature (Fan and Zhang, 2018). Essentially, male sterility of EGMS lines is stable within an environment (barring extreme weather events), but not across environments. Using this reversible sterile/fertile trait, EGMS lines can be produced in a two-line system. The EGMS line is grown in the sterility-inducing environment, where it is crossed with the inbred male parent to produce hybrid planting seed. To obtain new male-sterile seed, the EGMS line is grown in the fertility-permissive environment for seed increase (Chen and Liu, 2014). Because specific maintainer and restorer lines are not required for EGMS seed increase, there is a broader range of germplasm available for developing hybrids in the EGMS system than for CMS (Abbas et al., 2021). Examples of genes contributing to EGMS include *ZmTms5, OCL4*, and *dcl5* in maize and *Tms5* and *Tms10* in rice (**Supplementary Table 1**) (Zhou et al., 2016; Li et al., 2017; Yu et al., 2017; Teng et al., 2020; Yadava et al., 2021).

In rice and maize, genes associated with GMS are well-characterized, and hybrid production using male sterile lines is common (Li et al., 2007; Qi et al., 2020; Rout et al., 2020). In these crops, as well as in model organisms like *Arabidopsis*, substantial genetic resources exist regarding GMS that can be used for the discovery of homologous genes in other species. In this review, we summarized the current methods in genome editing that can be used for the generation of male sterile traits. We assessed the known biochemical modes of plant GMS and characterized them into four general groups: transcriptional regulation, splicing, fatty acid transport and processing, and sugar transport and processing. In addition, we explored protein sequence homology from EGMS genes of three monocots (maize, rice, and wheat) and three dicots (Arabidopsis, soybean, and tomato) and have identified 23 candidate genes within the upland cotton genome for the development of new male sterile germplasm to be used in cotton breeding. We also examined evolutionary relationships between monocot and dicot GMS genes to describe the relative similarity and relatedness of these genes.

## Genome editing strategies for generating male sterility

Various strategies can be used to introduce male sterility in a crop of interest. Historically, male sterile traits have been introduced *via* cross pollination to elite germplasm after observing naturally occurring male sterility within a species (Yamagishi and Bhat, 2014; Bruns, 2017; Zheng et al., 2020). This strategy was widely used before the advent of plant transformation; many of the well-known historical examples of crop male sterility such as cytoplasmic Texas male sterile maize and two-line hybrid rice were developed in this manner (Virmani, 2003; Bruns, 2017). A common drawback of this method is the long timeline needed for backcrossing the trait into elite germplasm – a process that takes multiple generations. Additionally, the discovery of natural male sterility in a breeding population or wild relative is oftentimes a chance event, and sufficient diversity may not exist or may not be visually observable in the species of interest. Traditional plant transformation (using *Agrobacterium* or the gene gun) has also been used to confer male sterility in crop plants. The development and adoption of genetic engineering techniques furthered the potential of developing male sterile lines and hybrid systems as breeders were no longer confined to the genetic variation available within the species of interest. Genetic engineering of male sterility meant that the gene of interest could be inserted directly into the elite line, but a reliance on tissue culture to recover mutants still makes this process time and labor-consuming. Additionally, plants engineered using *Agrobacterium* t-DNA systems are under strict regulations and the regulatory process adds considerable additional time to varietal release.

In recent years, CRISPR/Cas9 genome editing has revolutionized genetic engineering with its ability to precisely alter DNA using sequence-specific nucleases (Doudna and Charpentier, 2014). Briefly, the Cas9 protein is led to the target sequence in DNA using a homologous segment of RNA referred to as single-guide RNA (sgRNA) (Doudna and Charpentier, 2014). At the target sequence, the Cas9’s endonuclease activity causes double-stranded breaks (DSBs) in target DNA, predominantly triggering the error-prone non-homologous end joining (NHEJ) DNA repair pathway (Jiang and Doudna, 2017). NHEJ’s repair process joins the two broken ends of DNA together without using a template for repair, commonly causing random insertions and deletions (indels), leading to a knockout or knockdown of the target gene (Jiang and Doudna, 2017). Less frequently, the homology-directed repair (HDR) pathway is engaged, where a homologous segment of DNA is used as a repair template and precise insertions are made into the target sequence (Jiang and Doudna, 2017). The sequence-specific targeting of DNA is an improvement on *Agrobacterium* t-DNA and gene gun methods as the insertion will occur in a specific, predetermined location rather than randomly within the genome. In the United States, regulatory oversight is also less stringent for crops that are genome edited but do not contain transgenes when compared to crops genetically engineered with *Agrobacterium* (Entine et al., 2021).

Currently, the most well-defined and common use for CRISPR/Cas9 in plants is for performing gene knockouts with NHEJ. This makes the CRISPR/Cas system particularly useful in male sterility-related studies as the male sterile phenotype is frequently due to loss-of-function mutations in genes related to reproduction and fertility. For example, Jiang et al. (2021) identified 12 new GMS genes in maize by knocking out selected transcription factor genes using the CRISPR/Cas9 system. Although knock-ins with HDR are less often used due to the low-efficiency nature of this pathway in plant cells, HDR has been used in a few studies to introduce or entirely replace promoters for a gene of interest, referred to as “promoter swapping”; this method could be used to target promoters associated with male reproductive organs to induce male sterility (Čermák et al., 2015; Shi et al., 2017; Hummel et al., 2018; Li et al., 2020; Lu et al., 2021). This is a still developing area of study with great potential; however, no instances of promoter swapping have been reported in upland cotton.

**Figure 1:**
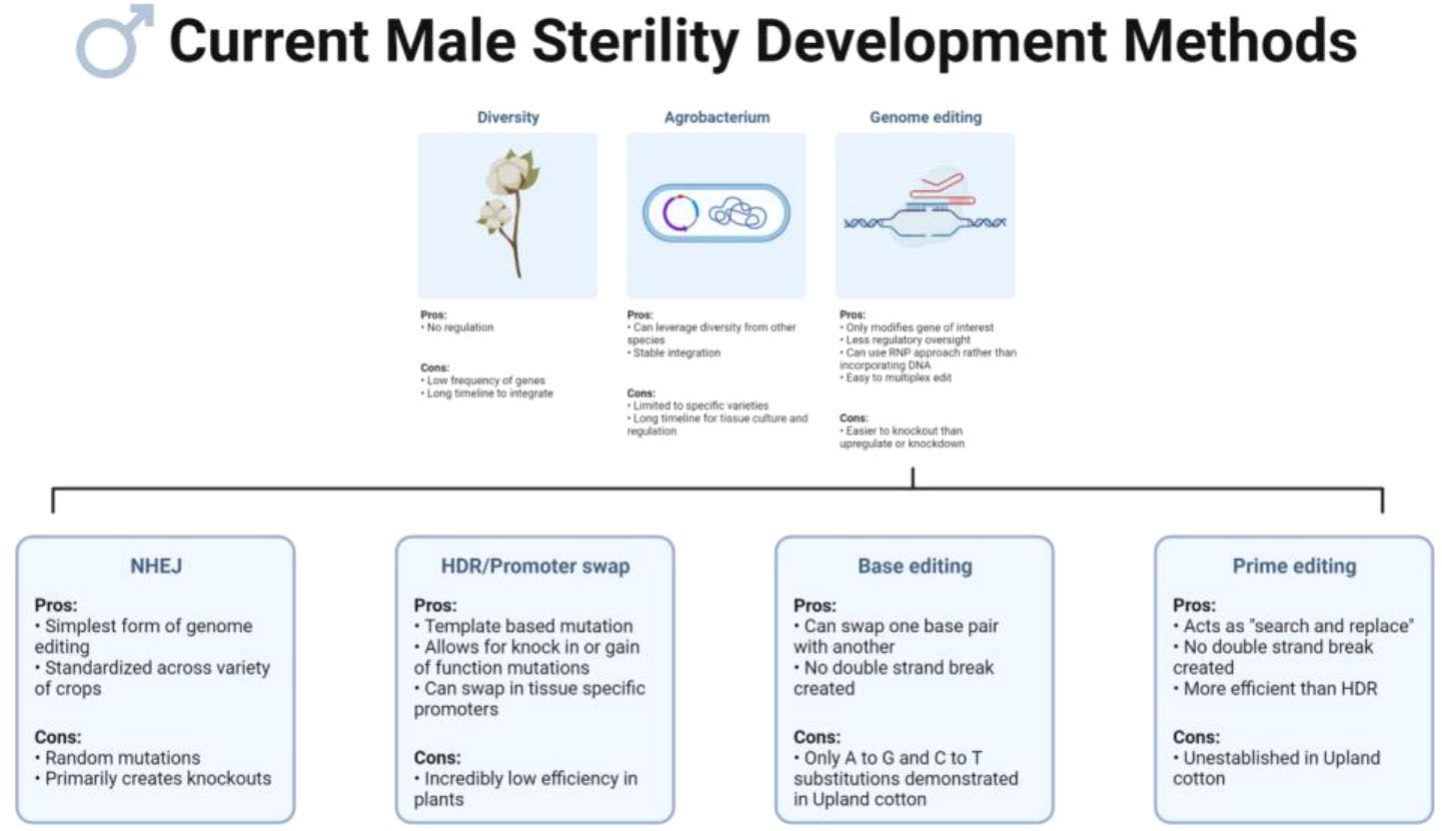
Current methods to develop male sterility in crops include making use of natural diversity, genetic engineering with *Agrobacterium*, and genome editing. Promising genome editing technologies include non-homologous end joining (NHEJ) for gene knockouts, homology dependent repair (HDR) for promoter swaps and gain-of-function mutations, base editing for amino acid substitutions, and prime editing for precise allele modifications. Created on biorender.com.

Other CRISPR/Cas system capabilities include multiplex editing, a commonly used strategy where multiple sites within a genome can be targeted at the same time by one CRISPR/Cas9 construct. Multiplex editing is typically used when attempting knockouts of multiple genes predicted to affect a trait, members of gene families, or simply to increase the chances of achieving the desired phenotype by targeting multiple sites within a single gene (Xie et al., 2015; Iqbal et al., 2016; Chilcoat et al., 2017; Abdelrahman et al., 2021). Base editing is another application of the CRISPR/Cas system which uses a Cas protein variant fused to a cytosine deaminase or adenine deaminase (Komor et al., 2016; Nishida et al., 2016; Gaudelli et al., 2017). In this system, specific substitutions (C to A, C to T, C to G, and A to G) can be directly induced in a target site without generating a DSB; this can be used to correct previous mutations, introduce premature stop codons to generate a knockout, or to produce a different amino acid than the one currently coded for in the DNA (Komor et al., 2016; Nishida et al., 2016; Gaudelli et al., 2017; Kim, 2018; Evanoff and Komor, 2019; Guyon-Debast et al., 2021). In upland cotton, successful base editing has been established for A to G and C to T substitutions, but at the present time other substitution types have not been reported (Qin et al., 2020; Wang et al., 2022). Prime editing, a more recent innovation in the CRISPR/Cas system originally developed in human cells, uses a modified Cas9 nickase-reverse transcriptase fused protein, paired with a prime editing guide RNA (pegRNA) for editing (Anzalone et al., 2019). Among the variations of the CRISPR/Cas system, prime editing is one of the most versatile and promising. Similar to base editing, prime editing creates mutations without the generation of a DSB, but, unlike base editing, prime editing can write new DNA sequences directly into the target genomic DNA and can create targeted insertions, deletions, and all twelve types of substitutions; this function has been dubbed “search and replace” (Anzalone et al., 2019; Lin et al., 2020; Marzec and Hensel, 2020). Prime editing has been tested in multiple crop and model species but has not yet been established in upland cotton (Jiang et al., 2020; Lin et al., 2020; Lu et al., 2021; Molla et al., 2021; Perroud et al., 2022). Some studies indicate that targeted insertions with prime editing are more efficient or comparable to HDR, but further optimizations will be necessary to fully adapt this technology to plants as editing efficiencies are still low (Lin et al., 2020; Molla et al., 2021). A summary of the pros and cons of each of these methods for developing cotton male sterility can be found in **Figure 1**.

One of the most important issues that must be accounted for when starting a CRISPR/Cas9 experiment is the recognition of possible off-target editing. Off-target editing often occurs when sgRNAs incorrectly target DNA sequences that are highly similar, but not identical, to the target sequence, leading to Cas9 cleavage and mutations in off-target locations (Modrzejewski et al., 2020). Compared to animal or human systems, Cas9 has highly specific activity in plants due to its the overall lower expression level, which reduces the occurrence of off-target editing (Peterson et al., 2016). Additionally, undesirable mutations can be eliminated through backcrossing in plants (Hahn and Nekrasov, 2019; Hajiahmadi et al., 2019). In addition to highly specific sgRNA design, various factors such as GC content of the target region, length of the sgRNA, number of mismatches proximal to the PAM site, plant tissue culture time, the type of Cas protein variant used and the associated promoter, and the fidelity of the available reference sequence, among others, have been shown to have effects on off-target activity, and are described in depth in recent reviews by Hajiahmadi et al. (2019) and Modrzejewski et al. (2020). However, generally speaking, the most important step in of avoiding off-target effects is first designing highly specific sgRNAs; if done well, off-target effects can be undetectable in the edited plant (Hajiahmadi et al., 2019). At present, multiple *in silico* prediction methods have been developed to streamline sgRNA design, and, while accounting for these factors, and suggest and rank the ‘best’ sgRNAs to use for a particular target (Haeussler et al., 2016; Alkan et al., 2018).

## Biochemical modes of pollen sterility

Although the control of male sterility can be divided between genes within the cytoplasm and the nucleus, the way these genes cause the sterility can broadly differ. To best review the broad classification of pathways these genes act in, four categories of functions were identified as common mechanisms: transcriptional regulation, splicing and long non-coding RNAs, fatty acid processing and transport, and sugar transport and processing. It is important to note that inclusion of a gene in one category does not mean that it will not impact other classes. Instead, genes were classified based on their direct mode of action while noting that they may cause downstream effects in other areas. These four categories were chosen with transcriptional regulators influencing the expression of other genes and pathways; splicing and long non-coding RNAs indicating genes that do not get translated into proteins but still have an impact on male sterility; and the final two categories indicating a gene preventing the proper formation or transport of a sugar or fatty acid.

### Transcriptional regulators

When considering the central dogma of biology, one of the most vital steps in the expression of a trait is the transcriptional regulation of the gene(s) controlling the trait. Many of the known genes controlling male sterility in crop species are classified as transcriptional regulators that up- or downregulate the expression of other genes. Often, a single gene will work to impact the expression of one or multiple genes, resulting in the change of phenotype. Examples of this include *Carbon Starved Anther* from rice which upregulates the expression of *MST8* and impacts sugar partitioning (Zhang et al., 2010); *Aborted Microspores* from Arabidopsis which causes premature tapetum and microspore degeneration (Sorensen et al., 2003); *ZmGAMYB/ZmGAMYB2* from maize which causes brown and shrunken pollen grains (Li et al., 2021); and *DYT1* from Arabidopsis which transcriptionally regulates 22 genes, with the knockout of *DYT1* preventing normal formation of the tapetum (Feng et al., 2012). Homology among plant species has been demonstrated for some of these genes including *ZmMs7*, where premature expression with an anther-specific promoter affects downstream genes, disrupting tapetum and pollen exine development. *ZmMs7* was originally discovered in maize but is homologous with *AtMs1* from Arabidopsis and *OsMs1* from rice (Zhang et al., 2018; An et al., 2020). *Baymax1* is a MYB transcription factor in rice which is conserved amongst many other species and causes male sterility by changing the expression of genes controlling tapetum and microspore development (Xiang et al., 2021). Although the focus of this paper is on male sterility, it is important to highlight that genes involved in transcriptional regulation can often show pleiotropic effects by impacting other traits, including biomass accumulation, a key trait contributing to crop yield (Mukhtar et al., 2018). Furthermore, it is possible that transcriptional regulators controlling male sterility have previously been unobserved in traditional mutation studies due to the quantitative nature of this trait. Recently, the advancement of genome editing technologies has aided in identifying novel genes impacting male sterility in maize through multiplexing the editing of several genes simultaneously. Among the 14 genes edited in a maize study by Jiang, et al., there was no male sterility displayed among single mutations, while some double and triple mutations caused sterility (Jiang et al., 2021). Furthermore, it is possible that the transcriptional regulation of a gene may only be observed in specific growing conditions, as shown with *TMS1* from Arabidopsis, which is overexpressed under heat stress to cause male sterility, or *Carbon Starved Anther* from rice, which causes photoperiod dependent male sterility (Yang et al., 2009; Li et al., 2022). Transcriptional factors are a key target for genome editing with many previously described genes in this classification preventing the proper development of pollen cells; however, when modifying transcriptional factors, it is important to recognize that they often interact with other genes and may cause undesired effects to traits that are unrelated to sterility and that the impact of these genes may change under varying environments.

### Splicing and long non-coding RNAs

Although the actual expression of a gene is incredibly important in impacting a phenotype, it is equally important that the gene is properly modified post-transcription. In plants, phased secondary small interfering RNAs (phasiRNAs) act as signals for cleaving specific mRNA sequences. PhasiRNAs can be separated based on their length as either 21 or 24 nucleotides (nt) in length. While 21-nt phasiRNAs were characterized in rice, 24-nt phasiRNAs were discovered in a variety of crops including rice, maize, wheat, litchi, orange, strawberry, and grape (Xia et al., 2019; Bélanger et al., 2020). Although 24-nt phasiRNAs were found in both monocots and dicots, each group underwent a different processing pathway for their development. Monocots have a unique version of Dicer, *DCL5*, which specifically contributes towards making 24-nt phasiRNAs, while dicots were thought to have 24-nt phasiRNAs produced by *DCL3* (Xia et al., 2019). Modification of *DCL5* resulted in temperature-dependent male sterility in maize, a beneficial trait to have as this removes the need for having a restorer line (Teng et al., 2020). Along with Dicer-like genes, *MEL1* in Arabidopsis and *Argonaute 18* are also involved in the creation of phased RNAs, with *Argonaute 18* causing a decrease in the stature of plants (Das et al., 2020; Lian et al., 2021). Mutations in the splicing signal of a gene can also cause significant changes in male sterility as observed with *PL1* from rice. The splicing mutation in *PL1* prevents proper formation of pollen exine, causing irregular shape to pollen cells and a lack of protection from stresses (Zou et al., 2020). Long non-coding RNAs may also contribute to male sterility with LDMAR shown to cause premature cell death in anthers under long day conditions (Ding et al., 2012). Long non-coding RNAs can be found in a broad range of species, acting in the modification of transcriptional messages. While these genes can impact sterility, they may also interact with alternative traits (Xia et al., 2019). As a result, when considering the editing of long non-coding RNAs, it is important to recognize that even if two genes appear to fall in the same family, they may not have the same impact across species.

### Fatty acid transport/processing

Another vital process for the stability of pollen cells is the processing and transport of fatty acids. Fatty acid chain length can be a critical component in the fertility of pollen cells as demonstrated by *ZmMs25, AtCUT1, OsHMS1*, and *OsGLI-4*. Expression of *ZmMs25* primarily occurs in the anther and it is thought this gene is involved in Acyl Co-A metabolism (Zhang et al., 2021c). Very long-chain fatty acids (VLCFAs) and their derivatives help maintain water content in pollen, an important component of pollen vitality. Mutations in these genes disrupt VLCFA biosynthesis or processing, leading to pollen desiccation and male sterility in dry environments. This produces a unique phenomenon in which male sterility is regulated by the humidity levels the plant is exposed to, as plants can still maintain pollen fertility under sufficiently humid environments (Millar et al., 1999; Yu et al., 2019; Chen et al., 2020). Although this trait does not allow for practical use in field-based conditions, it could be highly useful in controlled environment conditions to create a new source of controlling male fertility. Often, genes related to fatty acid transport and processing are vital in maintaining the integrity of the cell wall or membrane in pollen. Examples of this gene group include *OsDGD2β* (Basnet et al., 2019), *AtMS2* (Aarts et al., 1997), *NEF1* Arabidopsis (Ariizumi et al., 2004), and *OsNP1* (Chang et al., 2016). *OsNP1* has been further used in developing a transgenic sterility system in which a red fluorescent protein is used to separate transgenic fertile seeds from non-transgenic male sterile seeds (Chang et al., 2016). *Ms10* from rice is less studied; however, it likely functions in a similar manner to the genes described above as it causes a decrease in the exine waxy layer. In Arabidopsis, *AtFAX1* functions as a fatty acid exporter vital in ROS signaling and maintaining homeostasis throughout pollen wall development with a mutation in this gene causing sterility (Zhu et al., 2020). *Acos5* from Arabidopsis has proven to be another vital gene with its knockout preventing the formation of sporopollenin (de Azevedo Souza et al., 2009). When knocked out using genome editing, *OsACOS12*, a homolog of *ACOS5* in Arabidopsis found in rice, the pollen wall was unable to form correctly and caused male sterility (Zou et al., 2017). Homology between sterility genes can further be found in *defective pollen wall* from rice which is homologous with *ms2* from Arabidopsis and shifts fatty acid and alcohol content in pollen cells (Shi et al., 2011). *CYP703A3* works similarly in rice with mutants displaying small, pale anthers (Yang et al., 2018). Alongside genetics, environmental conditions could play a critical role in fatty acid regulation as CRISPR/Cas9 knockdown of *ACO2* in cotton resulted in male sterility when plants were grown in normal day and night temperatures, but plants grown in high day and night temperatures were fertile (Khan et al., 2020). Oftentimes genes involved in the processing and transport of fatty acids impact the stability of the pollen cell wall, with changes in function preventing pollen cells from retaining the appropriate amount of water. As a result, modifications in this class of genes can allow for an interesting range of effects depending on the environment, with humidity often being the largest controlling factor.

### Sugar transport/processing

Although transcriptional regulation, splicing, and fatty acid processing play a much larger part in the regulation of male sterility in plants, genes related to the processing and transport of sugar have also been shown an impact on the fertility of pollen. One example of this is *UGPase 1* in rice, where a SNP in this gene causes pleiotropic effects by increasing rice chalkiness and causing male sterility (Chen et al., 2007; Woo et al., 2008). Loss of *HXK5* in rice causes male sterility as pollen cells experience a decrease in starch content (Lee et al., 2019). This is further demonstrated in cucumber, where the downregulation of *CsSUT1*, a sucrose-proton transporter, leads to male sterility by altering sucrose, hexose, and starch supply available in pollen development (Sun et al., 2019). In Arabidopsis, *Thin Exine 2* (*TEX2* or *ROCK1*) functions as a nucleotide-sugar transporter, which when knocked out leads to the formation of an incredibly thin exine (Wang and Dobritsa, 2021). *Ms8* from corn is an example of a ß-1,3-galactosyltransferase which has been genome edited to create a transgene free, male sterile variety of corn (Chen et al., 2018). *ZmMs45* is another gene in maize which is thought to function as a strictosamide synthase and causes complete sterility when both copies are knocked out (Wu et al., 2016). When *ms39* or *callose synthase12* in maize is knocked out, the tapetum develops irregularly causing aborted microspores (Zhu et al., 2018). Finally, the regulation of genes surrounding carbohydrate metabolism are suggested to impact cytoplasmic male sterility in wheat; however, this has not been pinned to single gene impacts and thus remains largely hypothetical (Geng et al., 2018). Although the majority of genes contributing to male sterility can fit within the above categories, one which does not is *OsDEX1* which acts in calcium transport and homeostasis (Yu et al., 2016). Similarly, *OsFIGNL1* is a AAA-ATPase which causes problems in separation of chromosomes in pollen cells when mutated (Zhang et al., 2017). Elucidation of biochemical function of some male sterility genes still has not been completed including *GmMs1* from soybean and *ms2* from wheat (Ni et al., 2017; Jiang et al., 2021). Carbohydrate metabolism functions in a broad range of roles in the creation of male sterile lines, with some genes causing thin exines, others impacting the tapetum formation, and still others changing the carbohydrate availability in developing cells. Despite the broad range of knowledge known about male sterility, a significant amount of work is still needed in determining the biochemical functions of genes and how they may overlap across species.

## Homology and relationship between male sterility genes

### The need for gene identification from other crops

The sources of male sterility in a crop are practically limited with only a few genes in each species known to contribute to the fertility of pollen in a crop. In the past, methods for identifying genes related to male sterility have included genome-wide association studies (GWAS) and mutation analyses (Chen et al., 2007; Lafarge et al., 2017; Ma et al., 2021; Han et al., 2022). However, both methods require the phenotyping of hundreds of lines to identify genes controlling a trait for which variation may not exist in the population being studied. As a result, homology-based identification of genes controlling male sterility may offer an alternative approach to shorten the time necessary to create novel male sterile lines in economically important crops. A homology-based approach can be pursued through identification of known male sterility genes in a variety of crop species. As these genes are identified, a BLAST sequence similarity search is used to screen a variety of monocot and dicot species with the aim of finding genes with a high level of conservation amongst either monocots, dicots, or both.

The advent of genome editing in crops has allowed for rapid integration of gene knockouts in a variety of crops including rice, cotton, soybean, tomato, wheat, lettuce, and many other plant species (Shan et al., 2014; Belhaj et al., 2015; Long et al., 2018). In tomato, CRISPR-based genome editing has already been used to create novel sources of male sterility with the knockout of *SlSTR1*. The project took a transgenic approach when developing lines as a restorer gene was transformed into the tomato plants with genome editing elements (Du et al., 2020). Additionally, genome editing offers the capability to target specific genes and to differentiate amongst members of large gene families, an important consideration as genes like *Dcl5* can be members of gene families with a broad diversity of functions beyond male sterility (Teng et al., 2020). Furthermore, as genes controlling environmentally dependent male sterility show a range of responses depending on the variety, it is possible this trait may be quantitatively inherited (Teng et al., 2020). In order to overcome this complexity, genome editing allows for multiplexed knockout of many genes simultaneously allowing investigation of how genes impact sterility individually and in combination with one another (Minkenberg et al., 2017). Genome editing offers the opportunity to quickly mutate and screen dozens of genes for insertion into novel varieties and breeding schemes for use in crop improvement programs. Strategies utilizing genome editing can also be designed in such a way that the scheme will work across multiple species as demonstrated with the editing of *ms26* in corn, rice, sorghum, and wheat (Cigan et al., 2017). This homology based approach has already proven successful in rice where *TMS5* was mutated using CRISPR/Cas9 based on homology with maize *TMS5*, suggesting that our approach should be successful in cotton (Barman et al., 2019).

### Homology-based comparison of male sterility genes

**Figure 2:**
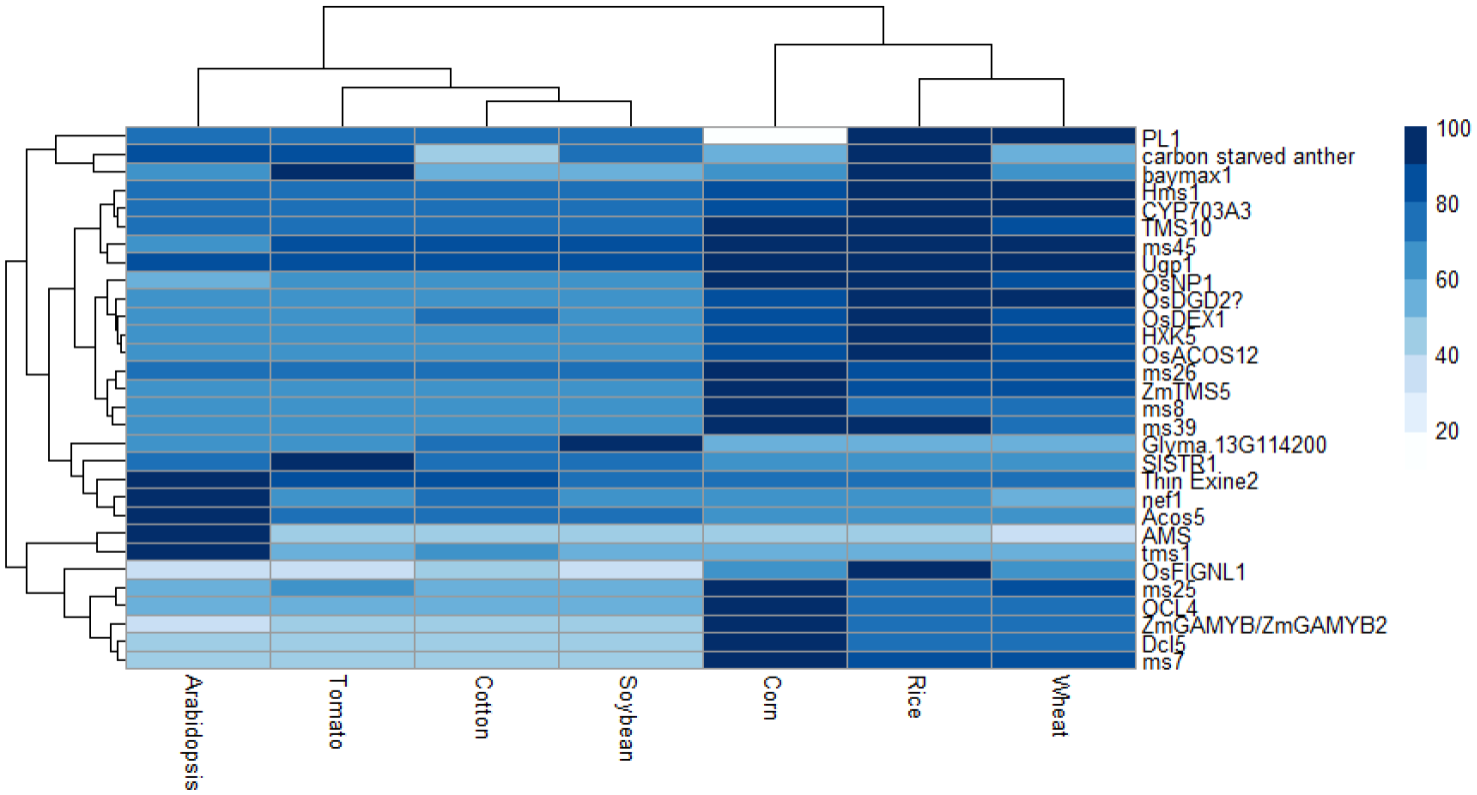
Heat map displaying the percentage of protein similarity between species. Dark blue is most similar and white is least similar.

Upon an extensive search for male sterility genes from diverse sources (**Supplementary Table 2**), sequences for these genes were obtained from databases including CottonGen (Yu et al., 2021), Sol Genomics Network (Fernandez-Pozo et al., 2015), RAP-DB (Sakai et al., 2013), TAIR (Berardini et al., 2015), SoyBase (Grant et al., 2010), and the Maize Genetics and Genomics Database (Woodhouse et al., 2021). A total of 38 genes were identified which contributed to male sterility; however, eight of these genes were excluded from subsequent analyses as they had not been finely mapped or had no corresponding protein. Sequences similar with each gene were searched in the cotton, soybean, rice, Arabidopsis, corn, wheat, and tomato genome databases to identify the level of sequence similarity for each gene (**Table 1**) as well as the protein it codes (**Figure 2**); this information is summarized in **Supplementary Table 2**.

**Table 1:** Homology-based comparison of thirty known male sterility genes*.

Lowly conserved	Monocot-specific	Dicot-specific	Highly conserved	Other
*OsFIGNL1*	*OCL4*	*SISTR1*	*Ugp1*	*Carbon starved anther*
*ZmGAMYB*	*ms7*	*Glyma.13g114200*	*OsACOS12*	*PL1*
*tms1*	*ms25*	*Thin exine 2*	*OsDEX1*	
*AMS*	*dcl5*	*ACOS5*	*Tms10*	
*Baymax1*		*nef1*	*CYP703A3*	
			*HXK5*	
			*Hms1*	
			*ms39*	
			*ms45*	
			*ZmTms5*	
			*ms26*	
			*ms8* *OsDGD2β* *OsNP1*	

*Genes lowly conserved have less than 50% homology across all species, monocot-specific greater than 70% homology among only the monocots, dicot-specific greater than 70% homology among only the dicots, and those that are highly conserved indicates greater than 70% conservation among all species. Genes not fitting in any of the four above categories are placed in the “Other” category.

By use of this method, genes identified can be classified into one of five categories: low conserved, in which only one species has the gene of interest, monocot-specific, dicot-specific, conserved among all species, or other. Among the queried genes, five are classified as low conserved, four as monocot-specific, five as dicot-specific, and 14 highly conserved among all species. As *carbon starved anther*, a gene discovered in rice, is conserved among dicot species except cotton but not among other monocot species, it is placed in the “Other” category. Additionally, the gene *PL1* is conserved among all species except maize, and thus is in the “Other” category (**Table 1**; **Figure 2**).

Utilizing candidate gene analysis from a homology perspective, the number of genes recommended for validation of their function within a novel species can be quickly narrowed down to make a starter list of candidate genes. For example, genes which are unique to one species may not be beneficial to pursue incorporation of them into another species as it is unlikely will have any impact on the new species of interest. On the other hand, genes conserved across a wide variety of species are likely to maintain the same function and cause similar phenotypes, so these types of genes may be good candidates. A final consideration to make when using homology-based candidate gene analysis is whether a gene is pleiotropic or not. In general, when trying to create a novel phenotype for incorporation into breeding programs, it would be most beneficial to modify only the trait of interest and, as a result, genes with pleiotropic effects should be avoided. In summary, the original list of 30 candidate genes was developed following the criteria: (1) reported genes that had already been mapped, (2) they had a protein sequence available, and (3) they did not have known pleiotropic effects. Among these, we prioritized genes that were conserved across all species based on our homology analysis.

To specifically search for candidate genes in upland cotton after the homology-based analysis, the 30-gene list acquired in the previous step (**Figure 2**) was narrowed down in a series of steps to a list of 23 genes (**Table 2**). Sequences from each gene were BLASTed against the TM-1 UTX v2.1 genome (Chen et al., 2020; Zhang, et al., 2015), and any genes that did not return a hit (*ms7*, *OsFIGNL1*) were discarded. Some genes from different species, for example, *ACOS5* from Arabidopsis and *OsACOS12*, identified a single ortholog in the TM-1 genome. *Ms25* from maize and *baymax1* from rice were another set of genes that were orthologous for a single gene in TM-1. Orthologous genes of this nature were consolidated to one candidate, further reducing the number of candidates. Next, we found orthologues for these genes in the TM-1 NAU-NBI genome to assess gene expression across tissue types using RNA-seq data (Dai et al., 2022); we presented these orthologues in **Supplementary Table 3**. Some genes that were present in the TM-1 UTX v2.1 genome were not present in the TM-1 NAU-NBI genome, so a few genes (*ZmGAMYB*/Z*mGAMYB2*, *Dcl5*, and *Glyma.13G114200*) were also discarded here, as we could not find expression data for these genes. Of this list of 23 candidates, *tms1*, *Zmtms5*, *OCL4*, and *TMS10* were TGMS genes, while *Hms1* and *carbon starved anther* were the only HGMS and PGMS genes, respectively. The remaining 17 candidate genes are not known to be environmentally sensitive or reversible male-sterile. This list of GMS genes in **Table 2**, as well as the expression data in **Supplementary Table 3**, provides a good starting point for researchers interested in hybrid cotton development, as well as basic research into cotton male sterility and reproduction in general.

**Table 2:** Twenty-three candidate genes identified for male sterility in upland cotton through homology-based analysis.

Source species	Gene	TM-1 UTX v2.1ortholog	TM-1 NAU-NBI v1.1 ortholog	Biochemical Mode of Sterility
Arabidopsis	*nef1*	Gohir.A01G020200.1	GH_A01G0145	Fatty acid transport/processing
Arabidopsis	*tms1*	Gohir.A09G166500.1	GH_A09G2429	Transcriptional regulator
Arabidopsis	*AMS*	Gohir.A11G063500.1	GH_A11G0588	Transcriptional regulator
Arabidopsis	*Thin Exine2*	Gohir.A11G201400.1	GH_A11G1840	Sugar transport/processing
Arabidopsis, Rice	*Acos5/OcACOS12*	Gohir.A03G125500.1	GH_A03G1091	Fatty acid transport/processing
Maize	*ZmTMS5*	Gohir.A03G065200.1	GH_A03G0574	Other (loss of RNase ZS1 function)
Maize	*ms39*	Gohir.A03G156600.1	GH_A03G1389	Sugar transport/processing
Maize	*ms8*	Gohir.A08G231100.1	Gh_A08G2286	Sugar transport/processing
Maize	*ms45*	Gohir.A09G144600.1	GH_A09G1354	Sugar transport/processing
Maize	*ms26*	Gohir.A12G164100.1	GH_A12G1506	Fatty acid transport/processing
Maize	*OCL4*	Gohir.A03G000036.1	GH_A03G1944	Transcriptional regulator
Maize, Rice	*ms25/baymax1*	Gohir.A09G129700.1	GH_A09G1215	Fatty acid transport/processing
Rice	*carbon starved anther*	Gohir.A03G101700.1	GH_A03G0883	Transcriptional regulator
Rice	*Hms1*	Gohir.A03G146400.1	GH_A03G1286	Fatty acid transport/processing
Rice	*OsDEX1*	Gohir.A09G163100.1	GH_A09G1518	Other (calcium transport and homeostasis)
Rice	*PL1*	Gohir.A10G187300.1	GH_A10G1679	Splicing
Rice	*TMS10*	Gohir.D11G055200.1	GH_A11G0480	Other (leucine-rich repeat receptor-like kinase)
Rice	*OsDGD2β*	Gohir.A11G086600.1	GH_A11G0785	Fatty acid transport/processing
Rice	*CYP703A3*	Gohir.A12G112600.1	GH_A12G1014	Fatty acid transport/processing
Rice	*OsNP1*	Gohir.A12G224500.1	GH_A12G2054	Fatty acid transport/processing
Rice	*HXK5*	Gohir.A13G196400.1	GH_A13G1742	Sugar transport/processing
Rice	*Ugp1*	Gohir.A11G169600.1	GH_A11G1563	Sugar transport/processing
Tomato	*SlSTR1*	Gohir.A09G212000.1	GH_A09G2313	Other (predicted: alkaloid biosynthesis)

## Conclusions and future perspectives

A homology-based analysis for the identification of target genes has clear benefits for the researcher, reducing the time and financial commitment required to perform large genetic screens, as is needed to perform GWAS. One of the major drawbacks of traditional genetic screens is their reliance on existing genetic variation to identify genes of interest. When using DNA or protein sequence homology as the basis for interspecific gene discovery, natural variation within a species is not required as we take advantage of the evolutionary conservation of genetic material. Targets can be identified even in species where sterile allele frequency is incredibly rare or phenotypic differences cannot be easily discerned. This is particularly relevant for male sterile traits as common phenotypes, such as differences in pollen shape or pollen shedding, can be subtle. Because this approach takes advantage of pre-existing resources and can be performed entirely *in silico*, the pipeline from identifying a trait of interest to generating multiple target genes is efficient.

In this study, we have demonstrated that protein sequence homology can be a basis for the discovery of many new target genes in upland cotton. Prior to this report, only a few confirmed sources of male sterility were previously known in this *Gossypium* species. These candidate genes now present promising targets for future genome editing efforts. Moreover, in crops where genetic resources are limited, such as orphan crops, this method can also be a cost-effective and time-efficient way to quickly identify new target genes and rapidly expand knowledge of crops’ genomes. In plant breeding, where the development of novel germplasm is an unabating need, identifying highly homologous genes among crop plants may facilitate new breeding strategies to capture hybrid vigor for improved crop productivity.

## Author contributions

KM, KH, JU, and JY conceived the study. KM and JY designed the study. KM and AB collected the data. KM, AB, MT, and JY analyzed the data. KM and AB drafted the manuscript. KH, JU, MT, and JY revised the manuscript. All authors contributed to the article and approved the submitted version.
